# Water binders in beef patties increase yield and extend shelf life

**DOI:** 10.1093/tas/txad091

**Published:** 2023-08-02

**Authors:** Jessie B Van Buren, Kendelle J Puga, Kacie C Hoffman, James A Nasados, Phillip D Bass, Michael J Colle

**Affiliations:** Department of Animal, Veterinary, and Food Sciences, University of Idaho, Moscow, ID 83844, USA; Department of Animal, Veterinary, and Food Sciences, University of Idaho, Moscow, ID 83844, USA; Department of Animal, Veterinary, and Food Sciences, University of Idaho, Moscow, ID 83844, USA; Department of Animal, Veterinary, and Food Sciences, University of Idaho, Moscow, ID 83844, USA; Department of Animal, Veterinary, and Food Sciences, University of Idaho, Moscow, ID 83844, USA; Department of Animal, Veterinary, and Food Sciences, University of Idaho, Moscow, ID 83844, USA

**Keywords:** beef patties, cook yield, shelf life, water binder

## Abstract

Identifying nonallergenic, natural water binders to increase beef patty juiciness and extend shelf life would be beneficial to the beef industry. The objective of this study was to determine the effect of integrating water binders into beef hamburger patties on cooking yield, shelf life, and pH. Five water binder treatments were added at 2% of the meat block. Treatments included potato extract, citrus fiber, dried refried beans, potato peel, or no binder (control). Six batches of each treatment were made and two patties from each batch were analyzed for each parameter. Fluid yield and lipid oxidation were measured on cooked, frozen (210 d), and reheated patties. Raw patties were used to evaluate color, fluid loss, and lipid oxidation over 4 d of retail display. Patties containing citrus fiber improved reheat yield (*P *= 0.03) and overall yield (*P *< 0.01). Citrus patties had the lowest pH (*P* < 0.01) at 5.45. On days 0 and 4 of retail display, patties containing a water binder treatment had less lipid oxidation than the control patties (*P* < 0.01). Additionally, the cooked, frozen, and reheated patties, had less lipid oxidation when containing a water binder treatment than the control patties (*P* < 0.01). Citrus fiber improved water retention in reheated patties, and all water binders delayed lipid oxidation in raw, cooked, frozen, and reheated patties. Increasing patty juiciness and delaying lipid oxidation will improve consumers’ eating experience of reheated, precooked patties in settings such as school or hospital cafeterias.

## Introduction

Precooked beef patties sold for food service settings such as schools and other institutions have increased fluid losses during reheating, decreased profits from lower yields, and drier patties due to a loss of water compared to raw patties (Huff-Lonergan and Lonergan, 2005). Additionally, cooked patties and patties at the retail case oxidize overtime leading to off-flavors and odors (Colle et al., 2019). The ability to maintain color, delay oxidation, and retain moisture could improve profits for the meat industry, decrease food waste, and increase palatability for consumers.

Water binders can be incorporated to keep beef patties palatable throughout frozen storage and reheating by increasing water retention to improve yields during cooking and potentially increase juiciness for consumers (Ray et al., 1981). The ability of a patty to retain moisture is also impacted by its pH, where nearing a pH of 5.2 decreases a protein’s water-binding ability (Huff-Lonergan and Lonergan, 2005). Binders commonly used in the industry include soy and sodium phosphate (Ray et al., 1981). Soy, however, is classified as an allergen (U.S. Food and Drug Administration, 2021), and sodium phosphate are considered an “un-natural” ingredient by consumers. Some ingredients that are added to patties can also act as antioxidants. Antioxidants are used to keep beef patties visually appealing during retail display by delaying oxidation to inhibit browning and the development of off-flavors and odors to extend shelf life (Ismail et al., 2009). Common antioxidants include tocopherol and ascorbic acid, which are also considered “un-natural” ingredients. The industry needs a natural, nonallergenic alternative to replace these water binders and antioxidants (Oswell et al., 2018).

Previous research has shown fruit and vegetable extracts may be possible alternatives (Kim et al., 2013; Gómez et al., 2016; Zhang et al., 2016; Colle et al., 2019; Pérez-Báez et al., 2020). Potato by-products have been shown to delay lipid and myoglobin oxidation while decreasing fluid loss and improving consumer sensory characteristics (Colle et al., 2019; Pérez-Báez et al., 2020). Additionally, citrus fiber has been shown to delay lipid oxidation in a retail setting (Fernández-López et al., 2004, 2005). However, there is a lack of research incorporating other possible natural binders into the meat. There is also a lack of evaluation of oxidation during storage at frozen temperatures of cooked beef patties, or analysis of the moisture loss during the reheating process. Changes during frozen storage and reheating need to be evaluated to understand the quality of the end product that will be consumed.

The objective of this study was to determine the effect of integrating water binders into beef hamburger patties on cooking yield, shelf life, and pH. Five water binder treatments were added at 2% of the meat block. Treatments included potato extract, citrus fiber, dried refried beans, and potato peel, or no binder (control).

## Materials and Methods

### Product Preparation

Beef chuck clod (USDA Choice; IMPS 114) was aged 14 d in refrigerated temperatures (2 °C) prior to grinding. The subprimals were coarse ground through a 0.9525 cm plate, and fine ground through a 0.3175 cm plate. The ground beef was analyzed and observed to contain 16% fat (Ground Beef Portable Fat Percentage Measuring Kit, Hobart Corporation, Troy, OH). Batches contained 4.5 kg ground beef, and 15% water, 1% salt, 0.2% onion granules, and 2% of the designated binder treatment. Ingredients were added as a percentage of the meat block. The five treatments included: control, citrus fiber (Citri-fi 125FG, Fiber Star Inc., River Falls, WI), potato extract (X-TRATOS, Item 207085, Basic American Foods (BAF), Blackfoot, ID), dried refried beans (Santiago, Item 67245, BAF), and potato peels (BAF). All binders were added in powder form. Dried refried beans and potato peels were ground using a coffee grinder (Adjustable Burr Grinder GX5000, Krups, Solingen, Germany) and filtered through an 80-mesh filter (Item 80BS8H, Advantech, Mentor, OH). Batches were mixed for 2.5 min at 29 rpm (DMX-50 Daniels 50 Lb. Mixer, Daniels Food Equipment, Parkers Prairie, MN). 1.59 cm-thick patties were formed and weighed 151.2 g each (Patty-O-Matic 220A patty former Patty-O-Matic Inc., Farmingdale, NJ). Batches were the experimental units with six batches of each treatment and two patties per analytical parameter.

### pH

Patty pH was measured on raw patties. A portable pH meter (Model SevenGo, Mettler Toledo, Woburn, MA) equipped with an InLab SolidsPro puncture-type electrode was used to measure pH. The pH meter was calibrated using standard pH 4.0 and 7.0 buffers.

### Cooking

Patties were cooked on a two-sided, clamshell-style electric grill (Cuisinart Griddler Deluxe Model GR-150) to a target internal temperature of 71 °C. Final internal temperature was recorded for each patty using hypodermic temperature probes (Omega Engineering Co., Stamford, CT) coupled with a 12-channel scanning thermocouple thermometer (Digi-Sense, Cole-Parmer Instrument Co., Vernon Hills, IL). Patties were frozen (−20 °C) individually on trays and stored in Great Value re-sealable freezer bags. After 210 d of frozen storage, patties were reheated as described previously. During cooking and reheating, patties from each treatment were placed in different grill locations to avoid potential differences in temperature.

### Cook Yields

Patties were weighed prior to being cooked and reweighed following 210 d of frozen storage and reheating to determine reheating yield and overall yield. Percent yield was calculated using the following equation:


$$\begin{array}{l} \%\;Yield=100-\left(\frac{Initial\;Weight-Final\;Weight}{Initial\;Weight}\times 100\%\right) \end{array}$$

### Cooked Lipid Oxidation

Thiobarbituric acid reactive substances (**TBARS**) were analyzed in duplicate following cooking, frozen storage, and reheating, following the protocol in Section XI, Appendix O of the Meat Color Measurement Guidelines (AMSA, 2012). Samples weighed 1 g. Briefly, samples were minced and duplicate 0.25 g samples were weighed into microcentrifuge tubes before 1.25 mL of the thiobarbituric acid stock solution was added. Samples were heated for 10 min in boiling water and then cooled in tap water. They were then centrifuged at 5,000 × *g* for 10 min in a microcentrifuge (Fisherbrand accuSpin Micro 17; Waltham, MA) for 10 min. Samples (200 µL) were then pipetted into a 96-well plate and absorbance was read at 532 nm using a microplate reader (Biotek Synergy 2, Santa Clara, CA).

### Retail Display

Patties used for retail display were placed on white Styrofoam trays, overwrapped with oxygen-permeable polyvinyl chloride film (oxygen transmission rate: 1,450 cc/645 cm^2^ per 24 h; water vapor transmission rate: 17.0 g/645 cm^2^ per 24 h; Koch Industries, Inc. #7500-3815; Wichita, KS), and placed in a retail display case (Model GDM-69, True Manufacturing Co., O’Fallon, MO) at 2 °C for 4 d. The display case was equipped with natural white Hg 40 W lights and had an average light intensity of 409 lux. To avoid potential effects due to display location, patties were rotated daily.

### Fluid Loss

Patties were weighed prior to being overwrapped and reweighed following 4 d of retail display to determine retail moisture loss. Percent retail fluid loss was calculated using the following equation:


$$\begin{array}{l} \%\;Retail\;Fluid\;Loss=\;\frac{Initial\;Weight-Final\;Weight}{Initial\;Weight}\times 100\% \end{array}$$

### Color

Retail display patties were allowed to bloom for 60 min, and two objective color measurements per patty were obtained using a HunterLab MiniScan EZ (Reston, VA) equipped with a 25-mm-diameter measuring area and a 10° standard observer. The instrument was set to Illuminant A and Commission Internationale de l´Eclairage L* (lightness), a* (redness), and b* (yellowness) values were recorded. Additionally, to evaluate differences in color change over time, Delta E was calculated using the following equation: Delta *E* = [(ΔL*)^2^ + (Δa*)^2^ + (Δb*)^2^]^0.5^ (King et al., 2023). Calibration of the instrument occurred by measuring against black and white calibration tiles prior to measuring color. Subsequent color measurements were taken daily on days 1, 2, 3, and 4. Discoloration (1 = very bright red, 8 = tan to brown) was measured daily by three evaluators following American Meat Science Association guidelines (AMSA, 2012). Evaluators were not color blind and were between 18 and 59 years old. Following screening, they were trained using pictures and in-person patties.

### Retail Lipid Oxidation

TBARS were analyzed in duplicate on days 0 and 4 of retail display as described above.

### Statistical Analysis

In this factorial design, there were four water binder treatments and one control treatment. Analyses collected at more than one time point were considered a split-plot design with repeated measures. Data were analyzed using mixed model analysis of variance. Water binder treatments, time (retail display or frozen storage), and their interaction were assumed as fixed effects. Time was considered a repeated measure modeled as a compound symmetric correlation structure. Subjective color scores were averaged across evaluators prior to analysis. Batch nested within treatment was considered a random effect. Cook yield data analysis used peak cook temperature as a covariate. Treatment least square means differences were assessed through pair-wise comparisons for significant effects. Significance was determined at *P* < 0.05. All statistical analyses were conducted using SAS V 9.4 (SAS Inc., Cary, NC).

## Results and Discussion

### pH

All patties containing water binders had different pH levels than the control patties (*P* < 0.01; Table 1). Average pH of the patties across treatments ranged from 5.45 to 5.56, which are within the standard range of beef pH (5.4 to 5.6; Huff-Lonergan and Lonergan, 2005). Patties containing dried refried beans, potato peels, and potato extract had the highest pH, whereas patties containing citrus fiber had the lowest pH. As proteins within meat approach the isoelectric point (5.2), their water-binding ability decreases (Huff-Lonergan and Lonergan, 2005; Aberle et al., 2012). In the current study, even though patties containing citrus fiber had the lowest pH, they were still within the normal range and far enough from the isoelectric point to negatively impact water holding capacity. The citrus fiber patties with a lower pH had higher yields during cooking.

**Table 1.** Estimated mean effects of water binding treatment on beef patty pH, reheat and cook yields, and fluid loss (*N* = 30)

Trait	Water-binding treatment^1^						
	Control	Dried refried beans	Citrus fiber	Potato peel	Potato extract	SEM	*P* value
pH	5.49^b^	5.56^a^	5.45^c^	5.56^a^	5.55^a^	0.01	<0.01
Reheat yield^2^, %	86.32^ab^	87.05^ab^	88.43^a^	84.68^b^	86.08^ab^	1.29	0.03
Overall cook yield^3^, %	59.76^b^	61.95^ab^	63.94^a^	60.75^b^	61.86^ab^	0.96	<0.01
Retail fluid loss^4^, %	0.82	0.65	0.73	0.90	0.70	0.22	0.90

^a,b,c^Within a row, means without a common superscript differ (*P* < 0.05).

^1^Treatments included no binder (control), dried refried beans, citrus fiber, potato peel, and potato extract added at 2% of the meat block.

^2^Reheat yield = 100 − [(frozen weight − reheated weight)/frozen weight * 100%].

^3^Overall cook yield = 100 − [(raw weight − reheated weight)/raw weight * 100%].

^4^Retail fluid loss = (day 0 weight − day 4 weight)/day 0 weight * 100%.

SEM = standard error of the mean.

### Cook Yields

Patties containing citrus fiber had higher reheat yields than the patties containing potato peels, but none of the treatments differed from the control patties (*P* = 0.03; Table 1). When evaluating the overall yield from raw patty to reheated patty, citrus patties had higher yields (*P* < 0.01; Table 1) than the control patties and patties containing potato peels. These results did not follow the hypothesized impact of pH on water-binding ability as previously stated above. However, Fernández-López et al. (2004) did find that citrus fiber had high water holding capacity similar to our findings. The citrus patty pH in the present study, although low, did fall within the normal range. Therefore, the increased yields could be due to the binder’s high fiber content. The poor yields of patties containing potato peels are likely due to the low starch content of the potato peel compared to the potato extract (Totosaus, 2009).

### Cooked Lipid Oxidation

**Figure 1.**
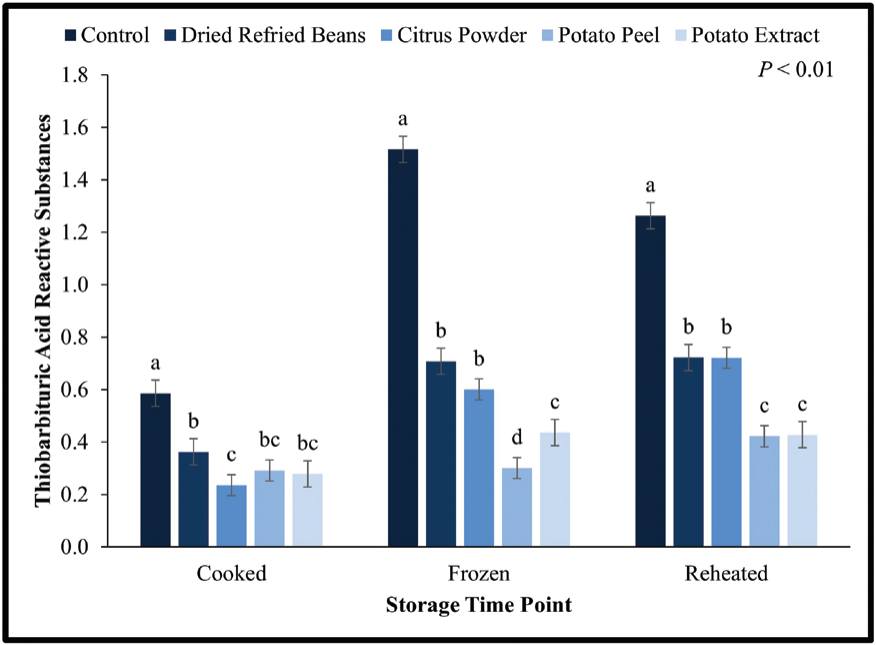
Lipid oxidation or thiobarbituric acid reactive substances (TBARS) values for water binding treatment by cooked storage time for beef patties. Each batch (*N* = 30) of patties was randomly assigned to be an untreated control or having a water binder incorporated at 2% of the meat block including dried refried beans, citrus powder, potato peels, and potato extract. Patties were cooked to 71 °C (Cooked), frozen at −20 °C for 210 d (Frozen), and reheated to 71 °C (Reheated). TBARS were measured following cooking, frozen storage, and reheating for the control and each treatment. Values are shown as least square means ± standard error. ^a,b,c^Within a time point, means without a common superscript differ (*P* < 0.05).

There was an interaction between cooked patty storage time and lipid oxidation (*P* < 0.01; Fig. 1). Following cooking, frozen storage, and reheating, the control patties consistently had greater lipid oxidation than patties containing water binders. It is important to note that control patties that had been through frozen storage and after reheating had more than 1.0 mg malondialdehyde/kg meat, which is the threshold for consumers to detect off-flavors in meat (Greene and Cumuze, 1982). Potato extract, potato peels, and pinto beans contain phenols that act as antioxidants (Akillioglu and Karakaya, 2010; Albishi et al., 2013). Similarly, citrus powder is high in ascorbic acid, a powerful antioxidant (Fernández-López et al., 2004). The authors hypothesize that the antioxidant activity in the by-products delayed oxidation of lipids.

When comparing individual treatment changes over time, control patties had the most lipid oxidation after frozen storage and the least after initial cooking. Patties containing dried refried beans and potato extract had the highest lipid oxidation after frozen storage and being reheated. Citrus patties had the most oxidation after reheating and the least after initial cooking. Patties containing potato peels had the most lipid oxidation after being reheated and the least after initial cooking and frozen storage. As expected, based on previous research, over time lipid oxidation levels increased, and applying heat increased the oxidation of lipids (Lawrie and Ledward, 2006).

### Fluid Loss

There were no differences between water-binding treatments in fluid loss during retail display of the raw patties (*P* = 0.90; Table 1). The average fluid loss across treatments was 0.76%. This is within the range of acceptable fluid loss of less than 2%, whereas a percentage fluid loss greater than 4% would start causing a loss in profits (Johnson, 1974).

### Retail Color

An interaction between day of retail display and water-binding treatment was observed for L* (lightness; *P* < 0.01; Table 2). On days 0, 1, and 3 patties containing potato peels were darker than all other treatments. Patties containing dried refried beans, citrus powder, or no binder, maintained lightness throughout the display. Patties containing potato peels lightened from days 0 to 4, and patties containing potato extract darkened after day 1. Additionally, an interaction between day of retail display and water-binding treatment was observed for a* (redness; *P* < 0.01; Table 2). Consistently, on each day of retail display patties containing potato peels were the least red. On day 4, patties containing dried refried beans and potato extract were the reddest. Consumers use color to determine freshness and prefer beef to be bright cherry red in color (Suman et al., 2014). Discoloration costs the beef industry $3.73 billion per year (Ramanathan et al., 2022). Therefore, utilizing new techniques to improve redness through 4 d of retail display is of high importance to the industry as it can decrease the potential for loss in profits due to discoloration. As expected, (Suman et al., 2014), all treatments significantly declined in redness each day of retail display. As oxymyoglobin is continuously exposed to oxygen, it oxidizes to metmyoglobin and decreases in redness (Suman and Joseph, 2013). An interaction between day of retail display and water-binding treatment was observed for b* (yellowness; *P* = 0.038; Table 2). On days 0 and 1 control patties and patties containing dried refried beans were yellower than the patties containing potato peels. On days 2 and 3 patties containing dried refried beans and potato extract were yellower than patties containing potato peels. Results in this study differ from previous research where potato peels increased b* values (Pérez-Báez et al., 2020). This may be due to differing pigmentation between the products. Further research is needed to eliminate the color pigment of the potato peel prior to its use in a retail setting. All treatments declined in b* values throughout retail display similar to a* values.

**Table 2.** Estimated mean effects of water-binding treatment and retail display time on beef patty objective color (*N* = 30)

Trait	Day of retail display	Water-binding treatment^1^					SEM	*P* value
		Control	Dried refried beans	Citrus fiber	Potato peel	Potato extract		
L*	0	47.20^a,z^	48.62^a,z^	48.09^a,z^	44.06^b,z^	48.06^a,y^	0.42	<0.01
	1	47.31^a,z^	48.85^a,z^	48.19^a,z^	44.47^b,yz^	48.12^a,y^		
	2	46.25^ab,z^	48.46^a,z^	47.36^a,z^	44.17^b,yz^	47.36 ^a,yz^		
	3	46.81^a,z^	48.38^a,z^	47.56^a,z^	44.40^b,yz^	46.92^a,z^		
	4	46.83^ab,z^	48.52^a,z^	47.84^a,z^	45.22^b,y^	46.78^ab,z^		
a*	0	24.35^a,v^	22.99^ab,v^	21.23^c,v^	17.33^d,v^	22.37^bc,v^	0.26	<0.01
	1	21.49^a,w^	20.50^a,w^	18.83^b,w^	14.85^c,w^	20.48^a,w^		
	2	18.52^a,x^	18.64^a,x^	16.97^b,x^	12.25^c,x^	18.65^a,x^		
	3	15.85^bc,y^	16.78^ab,y^	15.11^c,y^	10.53^d,y^	17.08^a,y^		
	4	13.61^b,z^	15.24^a,z^	13.75^b,z^	9.42^c,z^	15.38^a,z^		
b*	0	25.30^a,w^	25.20^a,w^	24.61^ab,w^	23.33^b,x^	24.41^ab,w^	0.29	0.04
	1	23.67^a,x^	23.73^a,x^	23.22^ab,x^	21.91^b,y^	23.40^a,x^		
	2	21.73^ab,y^	22.30^a,y^	21.77^ab,y^	20.50^b,z^	22.06^a,y^		
	3	20.73^ab,yz^	21.34^a,yz^	20.62^ab,z^	19.67^b,z^	21.35^a,yz^		
	4	19.88^a,z^	20.74^a,z^	20.14^a,z^	19.43^a,z^	20.51^a,z^		

^a,b,c,d^Within a trait and day, means without a common superscript differ (*P* < 0.05).

^v,w,x,yz^Within a trait and treatment, means without a common superscript differ (*P* < 0.05).

^1^Treatments included no binder (control), dried refried beans, citrus fiber, potato peel, and potato extract added at 2% of the meat block.

SEM = standard error of the mean.

There was a treatment difference for Delta *E* (*P* < 0.01) from days 0 to 4 of retail display (Table 3). The control Delta *E* was greater than all treatments which were not different from each other. Larger Delta *E* values indicate greater color change over the specified period of time (King et al., 2023). Therefore, in the current study, the lower Delta *E* values of the treated patties indicate that they were more stable through 4 d of retail display than the control patties. The antioxidants in the respective binders likely improved the color stability of the treated patties compared to the control.

**Table 3.** Estimated mean effects of water-binding treatment on delta color change from days 0 to 4 of retail display

	Water-binding treatment^1^					SEM	*P* value
	Control	Dried refried beans	Citrus fiber	Potato peel	Potato extract		
Delta *E*^2^	12.2^a^	9.1^b^	8.9^b^	9.1^b^	8.4^b^	0.4	<0.01

^a,b^Means without a common superscript differ (*P* < 0.05).

^1^Treatments included no binder (control), dried refried beans, citrus fiber, potato peel, and potato extract added at 2% of the meat block.

^2^Delta *E* = [(ΔL*)^2^ + (Δa*)^2^ + (Δb*)^2^]^0.5^.

SEM = standard error of the mean.

**Figure 2.**
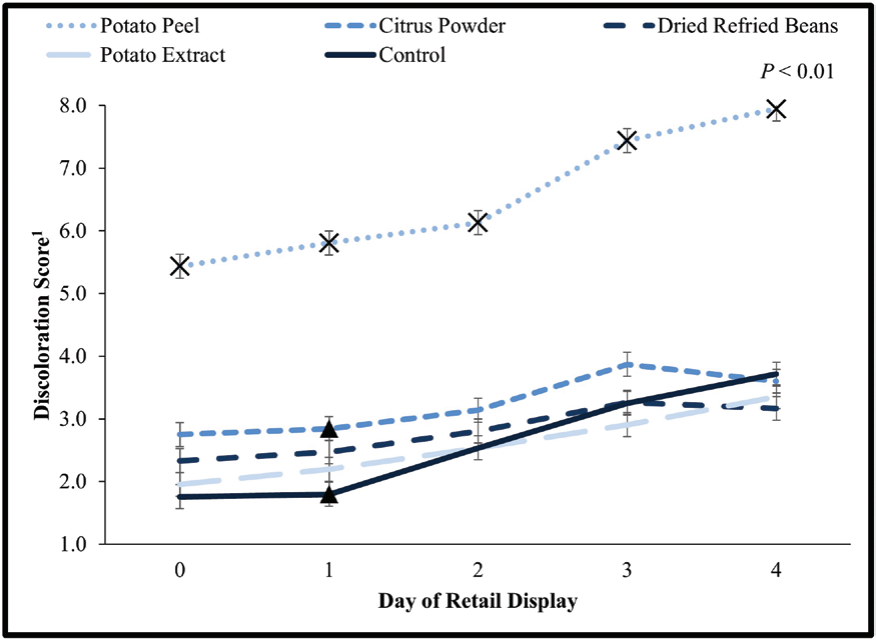
Subjective discoloration values for water binding treatment by retail display time for beef patties. Each batch (*N* = 30) of patties was randomly assigned to be an untreated control or having a water binder incorporated at 2% of the meat block including dried refried beans, citrus powder, potato peels, and potato extract. Patties were overwrapped with an oxygen permeable polyvinyl chloride film and displayed in a retail display case at 2 °C for 4 d. Discoloration was measured daily by three evaluators throughout retail display for the control and each treatment. Values are shown as least square means ± standard error. Multiplication symbol indicates that within a day, water binding treatment differs from all others (*P* < 0.05). Filled triangle indicates that within a day, means with a common shape differ (*P* < 0.05). ^1^Discoloration scale: 1 = very bright red, 8 = tan to brown.

**Figure 3.**
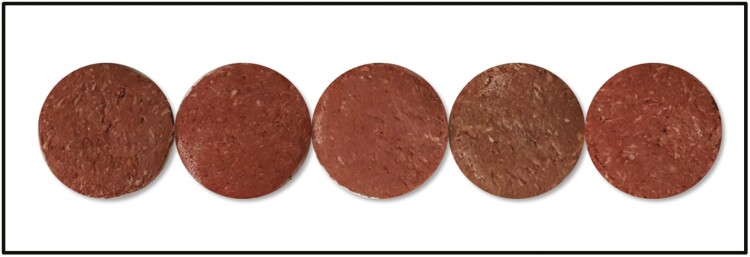
Beef patties on day 0 of retail display. From left to right: control (no binder), 2% dried refried beans, 2% citrus powder, 2% potato peel, and 2% potato extract. Patties were overwrapped with an oxygen permeable polyvinyl chloride film and displayed in a retail display case at 2 °C.

There was an interaction between day of retail display and water-binding treatment when evaluating patty discoloration subjectively (*P* < 0.01; Fig. 2). Patties containing potato peels consistently had the greatest discoloration each day of retail display (Fig. 3). Previous research has shown that potato peels will increase yellowness in raw meat due to the pigment with the peel (Pérez-Báez et al., 2020). Additionally, on day 1 patties containing citrus powder had a higher level of discoloration than the control patties. In general, within treatments patties increased in discoloration throughout retail display. The myoglobin within meat oxidizes as it is exposed to oxygen and the appearance of the meat discolors (Mancini and Hunt, 2005).

Based on color, potato peel would not be a suitable binder to use in beef patties. The low Delta *E* for the potato peel is likely due to the low initial L* and a* values observed on day 0 of retail display. These patties were dark, less red, and had greater discoloration than the control and other treatments throughout retail display.

### Retail Lipid Oxidation

**Figure 4.**
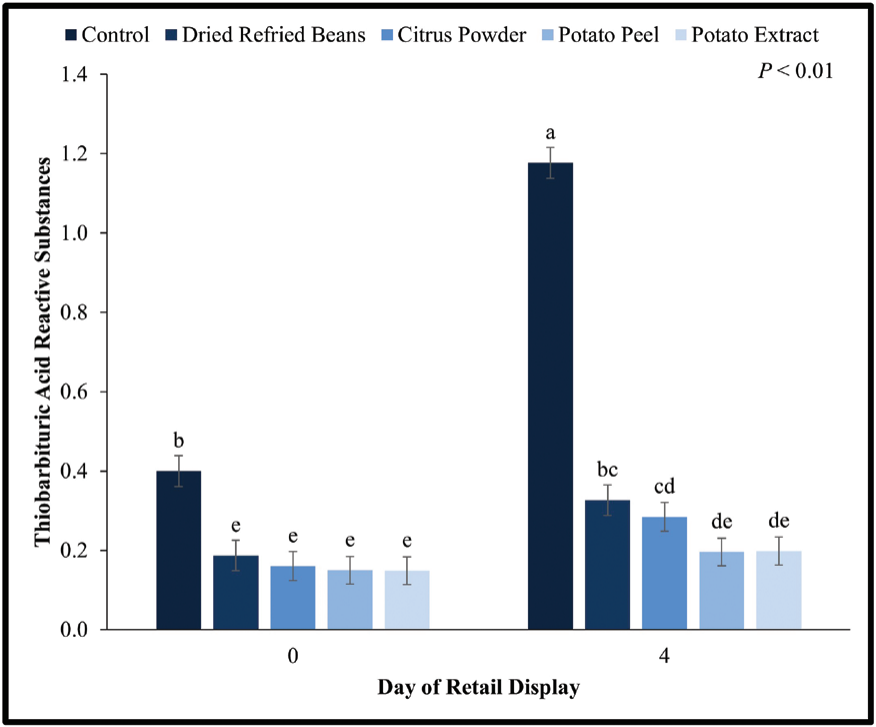
Lipid oxidation or thiobarbituric acid reactive substances (TBARS) values for water binding treatment by retail display time for beef patties. Each batch (*N* = 30) of patties was randomly assigned to be an untreated control or having a water binder incorporated at 2% of the meat block including dried refried beans, citrus powder, potato peels, and potato extract. Patties were overwrapped with an oxygen permeable polyvinyl chloride film and displayed in a retail display case at 2 °C for 4 d. TBARS were measured on days 0 and 4 of retail display for the control and each treatment. Values are shown as least square means ± standard error. ^a,b,c,d,e^Without a common superscript differ (*P* < 0.05).

There was an interaction between day of retail display and water-binding treatment for patty lipid oxidation (*P* < 0.01; Fig. 4). The control patties had greater lipid oxidation on days 0 and 4 than the patties containing water binders. Patties containing citrus fiber, potato peels, and potato extract had less lipid oxidation on day 4 than the control patties had on day 0. Furthermore, patties containing potato peels and potato extract did not increase in lipid oxidation from days 0 to 4. It is important to note that day 4 control patties were the only treatment to pass 1.0 mg malondialdehyde/kg meat, the threshold for consumers to detect off-flavors in meat (Green and Cumuze, 1982). These results are similar to the lipid oxidation results in the cooked patties as stated previously. Not only can the binders act as an antioxidant in cooked product, but also in raw products.

## Conclusion

The study provides evidence that incorporating citrus fiber into beef patties increases water retention to potentially improve juiciness and increase yields and profits for processors. Improving the quality of reheated beef patties could increase demand, while increasing yields and potential profits for meat processors. Additionally, the benefits of using by-products from dried refried beans, potato peels, citrus fiber, and potatoes were assessed and found to delay lipid oxidation to extend shelf life and inhibit the development off-flavors and odors in patties. Extending shelf life has the potential to decrease food waste both at home and at the retail setting. Further research must be conducted to determine the nutrient composition as well as sensory characteristics of patties made with the binders that improved cooking yield and shelf life. Specifically, citrus fiber, dried refried beans, and potatoes.
